# Health Tract No. 312

**Published:** 1868-03

**Authors:** 


HEALTH TRACT No. 312.

### EARLY RISING

Is a crime against the body and against nature unless it is preceeded by a proportionably early retiring. It is claimed for the French women who live in the large cities, who spend three fourths of their nights in parties, and balls, and dances, and the theatre, and the opera, retiring to their beds towards daylight, that they maintain their sprightliness, their vivacity and their good looks by the universal habit, and a strong determination arising from rational principles, that under all circumstances, after having retired to bed they will remain there until they have had their full sleep out, even if it requires until sun down. Our own experiences will always tell us that if insufficient sleep is had on any night it will be followed by a day of yawning, of discomfort, of disagreeable drowsiness and of inefficiency in whatever calling, profession or employment we may engage. All physiologists agree that the very first step towards madness is an insufficiency of sleep, whether compelled or voluntary. The babe gets fretful when it gets sleepy, and will remain fretful for hours, if its sleep is broken in upon. That same babe, if in good health, always wakes up of itself to crow and play and smile so covingly in a mother's eye. We can better and more safely intrench upon the necessary amount of food for ten days, than abate from the requisite amount of sleep for two, for the simple reason that the rest of good sleep recuperates the brain and the whole nervous system. An eminent biblical commentator thought to save time by rising at four in the morning, winter and summer; the result was an impairment of sight (by the sudden transition from the darkness of the closed eye, to the glare of artificial light) and general health, which required many months travel abroad, and enfeebled bodily health for the remainder of life, and this before he was three-score. The same custom of four o'clock rising followed by that sturdy old Roman, Josiah Quincy, brought its penalty along with it. Nature would not be cheated and the very moment he became inactively occupied he rapidly subsided into a nod. His son relates of him :

One day, Mr. John Quincy Adams, who was addicted to the same vice of intemperate early rising, with much the same consequences, was visiting my father, who invited him to go into Judge Story's lecture-room, and hear his lecture to his law class. Now, Judge Story did not accept the philosophy of his two friends in this particular, and would insist that it was a more excellent way to take out one's allowance of sleep in bed, and be wide awake when out of it—which he himself most assuredly always was. The Judge received the two Presidents gladly, and placed them in the seat of honor on the dais by his side, fronting the class, and proceeded with his lecture. It was not long before glancing his eye aside to see how his guests were impressed by his doctrine, he saw that they were both of them sound asleep, and he saw that the class saw it too. Pausing a moment in his swift career of speech he pointed to the two sleeping figures, uttered these words of warning: 'Gentlemen, you see before you a melancholy example of the evil effects of early rising!' The shout of laughter which followed, effectually aroused the sleepers. It is "early to bed" as well as "early to rise" which insures health, wealth and wisdom, provided a man goes to work when he does get up.

